# The Acute Relationships Between Affect, Physical Feeling States, and Physical Activity in Daily Life: A Review of Current Evidence

**DOI:** 10.3389/fpsyg.2015.01975

**Published:** 2015-12-23

**Authors:** Yue Liao, Eleanor T. Shonkoff, Genevieve F. Dunton

**Affiliations:** ^1^Department of Behavioral Science, The University of Texas MD Anderson Cancer CenterHouston, TX, USA; ^2^ChildObesity180, Tufts UniversityBoston, MA, USA; ^3^Department of Preventive Medicine, Institute for Health Promotion and Disease Prevention Research, University of Southern CaliforniaLos Angeles, CA, USA

**Keywords:** exercise, mood, accelerometry, experience sampling, ecological momentary assessment, free-living

## Abstract

Until recently, most studies investigating the acute relationships between affective and physical feeling states and physical activity were conducted in controlled laboratory settings, whose results might not translate well to everyday life. This review was among the first attempts to synthesize current evidence on the acute (e.g., within a few hours) relationships between affective and physical feeling states and physical activity from studies conducted in free-living, naturalistic settings in non-clinical populations. A systematic literature search yielded 14 eligible studies for review. Six studies tested the relationship between affective states and subsequent physical activity; findings from these studies suggest that positive affective states were positively associated with physical activity over the next few hours while negative affective states had no significant association. Twelve studies tested affective states after physical activity and yielded consistent evidence for physical activity predicting higher positive affect over the next few hours. Further, there was some evidence that physical activity was followed by a higher level of energetic feelings in the next few hours. The evidence for physical activity reducing negative affect in the next few hours was inconsistent and inconclusive. Future research in this area should consider recruiting more representative study participants, utilizing higher methodological standards for assessment (i.e., electronic devices combined with accelerometry), reporting patterns of missing data, and investigating pertinent moderators and mediators (e.g., social and physical context, intensity, psychological variables). Knowledge gained from this topic could offer valuable insights for promoting daily physical activity adoption and maintenance in non-clinical populations.

## Introduction

Despite the health-promoting effects of physical activity (Penedo and Dahn, 2005), most individuals are physically inactive in their daily lives (Bauman et al., 2011). A large body of research has suggested that physical activity can boost emotional well-being (i.e., the “feel good” effect; Reed and Ones, 2006). Several behavioral theories have suggested that individuals will engage in behaviors that provide pleasure (e.g., Bentham, 1962), or from which they anticipate a positive affective response (e.g., Mellers, 2000). Accordingly, people would be expected to engage in continued physical activity over time if they experienced a boost in positive affect but to exercise less across time if negative affect resulted. Yet, research is lacking on the extent to which these pleasure theories underlie individual motivations to engage in daily physical activity.

Until recently, most studies investigating the acute associations between affective states and physical activity have been conducted in controlled laboratory settings (e.g., performing pedaling exercise in a lab). Although lab studies allow researchers to have a precise control of the physical activity session (i.e., the intensity and duration), and an exact timing of when to assess affective states, the external validity of findings from these studies is questionable since both behaviors and emotion reactions could differ dramatically between lab-based and naturalistic settings (Gunes et al., 2008; Bussmann et al., 2009). One of the reasons is that in the laboratory, conditions are prescribed to participants, while in the real world, individuals have natural preferences and choices about situations they seek and avoid (Wilhelm and Grossman, 2010). Unveiling the acute relationships between affective states and physical activity in free-living situations might help to shed light on how people make decisions to engage in physical activity during their everyday lives. Moreover, most lab-based studies focus on affective responses either during or after physical activity. Whether affective states also act as precursors to engaging in physical activity is less clear. A better understanding of whether affective states predict physical activity (and vice versa) could have important implications for promoting everyday physical activity, especially long-term physical activity maintenance.

Previous studies on this topic usually use a variety of terms such as “affect,” “emotion,” and “mood” to describe people’s psychological experience. While psychologists have distinct definitions for each of these term (e.g., “emotion” is considered to be a more generic term than “affect” since it could include other attributes such as physiological changes; Lazarus, 1991), researchers from other fields (e.g., behavioral science and public health) sometimes use these words interchangeably. In addition, some physical activity researchers use the term “physical feeling states” to capture sensory experiences such as energy and fatigue that may be distinct from positive/negative affective states (Dunton et al., 2014). For this review, all terms used to describe the immediate emotional states that a person feels at any given moment were included, and were hereafter referred to as either “affective states” or “physical feeling states” to reflect their momentary nature.

The current review aimed to answer the following questions among non-clinical populations: (1) Do affective and physical feeling states predict subsequent physical activity levels in free-living situations? (2) Does free-living physical activity lead to improvement in subsequent affective and physical feeling states (e.g., increases in positive affect, decreases in negative affect)?

## Materials and Methods

### Literature Search Strategy

Literature searches were conducted for articles that were published in English using Medline, PsycINFO, and Google Scholar. Articles published prior to November 2, 2015 were included. The following keyword combinations were used: Physical Activity or Exercise and Mood or Emotion or Affect (see Supplementary Table S1 for sample full search strategy used for OVID Medline). Titles and abstracts identified through the search process were reviewed to identify relevant articles.

### Inclusion and Exclusion Criteria

Studies were included in this review that (1) examined the short term relationship between free-living physical activity and some type of affect or feeling measure; and (2) involved participants who were free from psychological- or physical-disorders. Free-living physical activity was defined as any physical activity that is not performed in a controlled environment or under extensive monitoring by research personnel for research study purposes.

Studies were excluded if: (1) the physical activity was performed in a controlled lab or research setting; (2) the time frame for the physical activity and affect relationship under examination was greater than 1 day; (3) affect was only measured during physical activity, or the temporal relationship between physical activity and affect was not clear (e.g., study only compared differences in affective state between active vs. non-active episodes); and (4) physical activity or affect were only assessed at one time point.

### Data Extraction

The data extraction procedure involved two stages. First, a coding form was developed for general data extraction to identify study characteristics including source, participants, methods, and results. Second, risk of bias assessment for each study was performed based on four domains including selection bias, confounders, data collection methods, and withdrawals and drop-outs, adapted after the Quality Assessment Tool for Quantitative Studies (National Collaborating Centre for Methods and Tools, 2008). Two reviewers performed the data extraction procedure independently. All discrepancies between reviewer’s ratings were resolved through discussions that led to a consensus.

## Results

### Literature Search

The initial searches yielded 3,745 potentially relevant articles. After title and abstract scanning, 211 articles were retrieved for further evaluation. One hundred and two studies were excluded because physical activity was not measured in a free-living situation. Forty-two studies were excluded because the association between physical activity and affect was observed over a time frame greater than 1 day. Twenty-eight studies were excluded because physical activity was only assessed at one time point (e.g., one aerobic class session). Another 25 studies were excluded because the temporal precedence of either physical activity or mood was not clear (e.g., examined the association between average daily physical activity level and average daily mood) or because of the cross-sectional study design. A total of 14 unique studies were eligible for the final review (see Supplementary Figure S1).

### Study Characteristics

**Table 1** summarizes the general characteristics of the 14 included studies. A majority of the studies (*N* = 12) utilized an experience sampling or Ecological Momentary Assessment (EMA) method (for more details about these two methods, see papers by Scollon et al., 2003; Shiffman et al., 2008, and Ebner-Priemer and Trull, 2009). Among these studies, 9 used electronic devices (e.g., a PDA) to deliver surveys and collect responses, and the other three used paper-pencil surveys with beepers to signal alerts. Only 6 studies used an objective measure (i.e., accelerometer) of physical activity. Seven studies used a self-reported physical activity instrument. One used gym attendance (based on a computerized scan of membership card for gym entry) as a proxy for physical activity and cross-checked with self-reported exercise tracking forms.

**Table 1:** Characteristics of included studies.

First author, year	Country	*n*‡	Participant characteristics	Mean age (range)	Female %	Affect assessment	Affect assessment frequency	Physical activity assessment
Kanning et al., 2015	Germany	69	A randomized sample of older adults	60.1(50–70)	51%	A six-item short scale adapted from the Multidimensional Mood Questionnaire (measures energetic arousal, valence, and calmness, with two bipolar for each).	When accelerometer detected a volume of physical activity that surpassed a predefined activity threshold (activity >220 mili-g; 10-min moving average) or fell below a predefined inactivity threshold for 3 days.	Activity level measured from accelerometer for 3 days.
Dunton et al., 2014	United States	119	Children from low to middle income households	N/A(9–13)	51%	Rating on eight adjectives assessing PA (two items), NA (four items), energy (one item), and fatigue (one item).	Randomly 3–7 times a day during preprogrammed intervals for 4 days.	Activity level measured from accelerometer for 4 days.
von Haaren et al., 2013	Germany	29	Inactive college students	21.3(N/A)	N/A	A six-item short scale adapted from the Multidimensional Mood Questionnaire (measures energetic arousal, valence, and calmness, with two bipolar for each).	Randomly every 2 h between 10 am and 10 pm for 2 days.	Activity level measured from accelerometer for 2 days.
Guérin et al., 2013	Canada	63	Active mothers	42.6(N/A)	100%	PA subscale (10 items) from the Positive and Negative Affect Scale (PANAS).	Before, after, and 3-h after each self-reported moderate-to-vigorous physical activity session for 2 weeks.	Self-reported type, intensity, and duration of exercise.
Kanning, 2013	Germany	87	College students	24.6(N/A)	54%	A six-item short scale adapted from the Multidimensional Mood Questionnaire (measures energetic arousal, valence, and calmness, with two bipolar for each).	Randomly every 45 min for 14 h.	Activity level measured from accelerometer for 1 day.
Kanning et al., 2012	Germany	44	College students	26.2(N/A)	48%	A six-item short scale adapted from the Multidimensional Mood Questionnaire (measures energetic arousal, valence, and calmness, with two bipolar for each).	Every 45 min for 14 h.	Activity level measured from accelerometer for 1 day.
Mata et al., 2012	United States	53	Volunteers recruited from community	25.4(N/A)	70%	Rating on 11 adjectives assessing PA (four items) and NA (seven items) guided by PANAS.	Every 90 min (up to eight times each day) for 7–8 days.	Self-reported activity level (Godin) at each electronic survey prompt.
Wichers et al., 2012	Belgium	504	Female twins from the general population	27(18–46)	100%	Ratings on 10 adjective assessing PA (four items) and NA (seven items) guided by PANAS.	Randomly every 90 min (up to 10 times each day) for 5 days.	Self-reported one item of activity level on a 7-point Likert scale at each electronic survey prompt.
Schwerdtfeger et al., 2010	Germany	124	Volunteers recruited through campus	31.7(18–73)	52%	Rating on 11 adjectives assessing PA (six items) and NA (five items).	Every 1 h for 1 day (about 12 total for each participant).	Activity level measured from accelerometer for 1 day.
LePage and Crowther, 2010	United States	54	Regularly active college students	19.1(N/A)	100%	PA (10 items) and NA (10 items) subscales from the PANAS – Expanded Form (PANAS-X).	Randomly four times a day and following each self-reported exercise bout for 10 days.	Self-reported type and amount of exercise.
Dunton et al., 2009	United States	23	Inactive adults aged 50+ years recruited from community	60.7(50–76)	70%	Rating on 10 adjectives assessing PA (one items), NA (seven items), energy (one item), and fatigue (one item).	Four fixed times (7:45 am, 11:45 am, 3:45 pm, and 7:45 pm) per day for 2 weeks.	Self-reported type and duration of exercise.
Carels et al., 2007	United States	36	Obese participants recruited from community	49.3(N/A)	89%	A single-item, unidimensional 10-point feeling scale ranging from “very negative mood” to “very positive mood.”	Daily diary filled out each morning and before bedtime, and before and after each self-reported exercise bout for 8 weeks.	Self-reported type, intensity, and duration of exercise.
Annesi, 2002	United States	69	Fitness center members from community	37.9(21–60)	71%	Exercise-induced Feeling Inventory (12-item) assessing positive engagement, revitalization, tranquility, and physical exhaustion.	Before and after each self-reported exercise bout for 6 weeks across 14 weeks.	Fitness center attendance cross-checked with self-reported exercise tracking forms.
Gauvin et al., 1996	United States	86	Recruited through YMCA from community	32.9(N/A)	100%	Exercise-induced Feeling Inventory (12-item) and 4 adjectives for PA, 5 adjectives for NA.	Randomly four times a day, and before and after self-reported exercise bouts for 6 weeks.	Self-reported intensity of physical activity (that lasted at least 20 min).

### Quality Assessment

**Table 2** shows the results of the quality assessment for each study. Selection bias was generally high (rating of weak) since most studies used convenience samples (e.g., college students) instead of randomly selected participants from the general population (only one study recruited this way). All studies used appropriate statistical methods (e.g., multilevel modeling) to control for clustering within individuals and some studies additionally controlled for other within-person confounding variables. Data collection methods were rated for both affect and physical activity assessment, and one studies received a weak rating due to the use of unestablished assessment tools. Three studies received a weak rating on withdrawals since they did not report any information regarding study drop-outs or missing data. Overall, three studies received a strong global rating and three studies received a weak global rating.

**Table 2:** Quality assessment of included studies.

Study	Selection bias	Confounders^1^	Data collection methods^2^	Withdrawals and drop-outs	Global rating^3^
Kanning et al., 2015	Moderate	Strong	Strong	Strong	Strong
Dunton et al., 2014	Weak	Moderate	Strong	Strong	Moderate
von Haaren et al., 2013	Weak	Moderate	Strong	Weak	Weak
Guérin et al., 2013	Weak	Moderate	Strong	Strong	Moderate
Kanning et al., 2013	Weak	Moderate	Strong	Strong	Moderate
Kanning et al., 2012	Weak	Moderate	Strong	Weak	Weak
Mata et al., 2012	Moderate	Strong	Strong	Strong	Strong
Wichers et al., 2012	Strong	Strong	Moderate	Strong	Strong
Schwerdtfeger et al., 2010	Weak	Strong	Strong	Weak	Weak
LePage and Crowther, 2010	Weak	Moderate	Moderate	Strong	Moderate
Dunton et al., 2009	Weak	Moderate	Moderate	Strong	Moderate
Carels et al., 2007	Moderate	Strong	Weak	Moderate	Moderate
Annesi, 2002	Weak	Moderate	Moderate	Strong	Moderate
Gauvin et al., 1996	Weak	Moderate	Moderate	Strong	Moderate

*^1^Studies were rated as strong on confounders (i.e., sufficiently controlled for potential confounding variables) if a multilevel modeling method was used (i.e., controlling for multiple observations within-person) and other within-person confounding variables were included (e.g., activity taking place 15 min before the affect assessment, pre-activity affect and feeling states, pleasantness of activity, and time of day). Studies were rated as moderate if only one of the two methods of controlling for confounding was used. ^2^Affect assessment and physical activity assessment were rated separately for data collection methods for each study. Studies were rated strong for affect assessment if they used an established scale with known reliability and validity. Studies were rated as strong for physical activity assessment if an objective measure was used (e.g., accelerometer);as moderate if a valid and reliable self-reported measure was used; and as weak if no validity or reliability of the self-reported measure was provided. ^3^Studies were rated as strong if they received three Strong ratings with no Weak ratings; as moderate if less than three Strong and one Weak ratings; as weak if two or more Weak ratings*.

### Short-Term Associations of Affective States with Subsequent Physical Activity

Only 6 of the 14 studies examined whether affective states predicted subsequent physical activity. Three studies found a significant positive association between positive affect and subsequent physical activity (Carels et al., 2007; Dunton et al., 2009; Schwerdtfeger et al., 2010), two found positive but non-significant association (Mata et al., 2012; Dunton et al., 2014), and one found a significant negative association (Wichers et al., 2012). In these studies, the length of accumulated physical activity levels being examined ranged from 30 min after assessment of positive affect (Schwerdtfeger et al., 2010; Dunton et al., 2014) to the whole day after affect assessment in the morning (Carels et al., 2007). Overall, based on findings from these studies, there is preliminary evidence for positive affective states predicting subsequent physical activity.

Five studies examined the association between negative affect and subsequent physical activity. One study found that higher negative affect was associated with less physical activity within a subsequent 30-min window (Dunton et al., 2009). None of the other four studies found a significant association (Schwerdtfeger et al., 2010; Mata et al., 2012; Wichers et al., 2012; Dunton et al., 2014). Therefore, preliminary evidence suggests that negative affective state is not necessarily an antecedent of physical activity in free-living situations.

Two studies also examined the association between physical feeling states (i.e., energy and fatigue) and subsequent physical activity. One study found that feeling more energetic led to more subsequent physical activity, and feeling more tired was associated with less subsequent physical activity (Dunton et al., 2014) in children. The other study did not find any significant association among middle-aged to older adults (Dunton et al., 2009). Overall, the evidence for physical feeling states predicting subsequent physical activity is very limited and inconclusive.

### Short-Term Associations of Physical Activity with Subsequent Affective States

Twelve studies examined the effects of physical activity on subsequent affective state. Eight out of the 11 studies found a significantly positive association between physical activity and subsequent positive affective state. In these studies, the time frame of the association ranged from immediately after (Gauvin et al., 1996; Carels et al., 2007; LePage and Crowther, 2010), to 3 h after a physical activity bout/session (Guérin et al., 2013). Three studies did not find a significant relationship (Mata et al., 2012; von Haaren et al., 2013; Kanning et al., 2015), and one did not report the significance test result (Annesi, 2002). Thus, based on these results, there is some consistent evidence for physical activity improving subsequent positive affective state.

Of the five studies that tested the effects of physical activity on subsequent negative affective state, two found a significant decrease in negative affect after engaging in physical activity (Gauvin et al., 1996; LePage and Crowther, 2010), two found no significant association (Mata et al., 2012; Wichers et al., 2012), and one found no significant association at within-person level but a significantly negative association at between-person level (Dunton et al., 2014). Therefore, the current evidence for physical activity decreasing subsequent negative affective state is mixed and inconclusive.

Seven studies also tested effects of physical activity on subsequent physical feeling states (e.g., energetic and calmness/tranquility). Five studies found a significant positive association between physical activity and subsequent feeling of energy (Gauvin et al., 1996; Kanning et al., 2012, 2015; Kanning, 2013; Dunton et al., 2014), one did not find a significant association (von Haaren et al., 2013), and one did not report the significance test result (Annesi, 2002). Thus, there is some evidence for physical activity increasing subsequent feeling of energy. Three studies found a significant decrease in calmness (Kanning et al., 2012, 2015; Kanning, 2013), one found a significant increase (Gauvin et al., 1996), one did not find a significant association (von Haaren et al., 2013), and one did not report significance test result (Annesi, 2002). Thus, the effect of physical activity on subsequent feelings of calmness is mixed and inconclusive.

## Discussion

To the authors’ knowledge, this review is one of the first attempts to systematically examine the bi-directional acute relationship between affective and physical feeling states and free-living physical activity among non-clinical populations. The fact that only a limited number of studies were included in our review suggests that more research is needed to better understand the links between affective states and free-living physical activity, especially whether and how affective and physical feeling states might act as a predictor for daily physical activity. Findings from this review show that positive affective states could potentially lead to being more physically active subsequently, although this positive relationship did not always reach significance. Further, most studies did not find a significant relationship between negative affective state and subsequent physical activity. Physical feeling states (i.e., energy and fatigue) might also be a predictor for free-living physical activity, although current evidence only showed this effect among children but not older adults. Overall, current literature suggests that people’s affective states, especially positive affective states (e.g., happy, excited), might be a predictor of free-living physical activity. Similar to the findings from lab studies (e.g., Reed and Ones, 2006), results from this review show that physical activity seems to improve immediate subsequent positive affective states and enhances feelings of vitality in people’s daily lives. However, engaging in physical activity may not necessarily decrease subsequent negative affective states (e.g., feeling stressed, sad, anxious) among non-clinical populations.

### Limitations of Current Studies

The present review used a modified version of Quality Assessment Tool for Quantitative Studies to assess study quality across all included studies, which addressed aspects (e.g., selection bias, data collection methods, withdrawals/missing data) that could be important for future studies to consider when investigating the acute relationships between affective and physical feeling states and physical activity in free-living settings. First, most of the reviewed studies used a convenience sample (e.g., undergraduate students from a Psychology class). Therefore, findings from these studies might not be representative of the general population, or high-risk populations for physical inactivity. Future studies on this topic would be strengthened by recruiting more representative (e.g., diverse age, ethnicity, and socioeconomic background) study participants from community-based samples.

Secondly, only six studies used both an electronic device to deliver and record momentary mood assessment, and an accelerometer to measure physical activity levels; which is considered as the highest methodological standard to use for investigating within-person associations between momentary affective states and physical activity in everyday lives (see the position statement by Kanning et al., 2013). Further, in order to capture a more representative sample of people’s behaviors, the total monitoring period should also be longer (e.g., more than 1 day).

Finally, none of the reviewed studies that used electronic devices provided information regarding level of data loss or missing data. As reported by Dunton et al. (2012), missing data from EMA could be due to non-compliance (i.e., non-response or non-wear) by the participants, or could be due to technical problems with the devices (e.g., device lost/damage, battery drain, app failed to initiate). A better understanding of the reasons for data loss could provide important insights for future studies that may want to adopt technology-based devices (e.g., whether researchers should put more effort into ensuring participant compliance or enhance device reliability). Therefore, when reporting results, researchers should consider providing such information.

### Future Directions

Future research in this area could be informed through the exploration of potential moderators of the acute relationships between affective states and physical activity, such as co-occurring contextual exposures. Studies have suggested that adults are more likely to be physically active if their significant others are also exercising with them (Giles-Corti and Donovan, 2002), and are less active when they are at home indoors compared with other locations (Liao et al., 2015). Further, being with other people may enhance positive affective response during physical activity compared to alone (Dunton et al., 2015), and more positive affective states occurred when outdoors and with others than when indoors and alone (Dunton et al., 2011). More studies on this topic are needed to examine the multi-way interactions among physical activity, affective, and physical feeling states, and physical and social contexts to find an optimal contextual setting for physical activity engagement. In addition, other psychological variables could influence both affective/physical feeling states and physical activity levels. For instance, one of the reviewed studies showed that the more the physical activity was autonomously regulated, the more energetic individuals felt afterwards (Kanning et al., 2012), and the other study found that higher momentary self-efficacy led to a higher physical activity level (Dunton et al., 2009). It would be worthwhile to investigate how fluctuations in these psychological variables might acutely influence affective states and physical activity or moderate their relation. For example, does positive affective response to physical activity change global self-efficacy or outcome expectancies over time, leading to more physical activity?

Further, intensity and duration of physical activity may influence the affective responses to physical activity (e.g., the dual-mode model; Ekkekakis, 2005). However, research in this area has mainly been performed in controlled laboratory settings. Only two of the reviewed studies examined the role of perceived intensity and both found that higher intensity activities led to a greater increase in positive affect afterwards (Carels et al., 2007; Guérin et al., 2013). In addition, since sedentary behavior is associated with increased risks of metabolic syndrome and cardiovascular disease (Edwardson et al., 2012; Wilmot et al., 2012); and there are studies demonstrating the metabolic-health benefits from breaks in sedentary time (e.g., some light activity in-between sedentary time; Owen et al., 2010). It would be also be a promising direction to examine how reduced sedentary time may affect affective states in future studies.

Lastly, although observational studies carried out in free-living settings have the strengths of demonstrating ecological validity and temporal precedence of the associations between affective states and physical activity, these studies are limited in their ability to draw causal conclusions. Future studies should seek out methods to test these associations using experimentally based approaches such as randomly assigning individuals to affect or activity conditions and measuring subsequent free-living behavior.

In summary, more research is needed to elucidate the bi-directional relationships between affective and physical feeling states and physical activity, especially whether and how affective and physical feeling states might acutely predict subsequent activity in people’s everyday lives. More research in these areas would be valuable for physical activity adoption and long-term physical activity maintenance strategies.

## Author Contributions

YL conceived of the study, conducted the literature search, performed data extraction, and drafted the manuscript. ES performed data extraction. GD conceived of the study, participated in its design, and helped to draft the manuscript. All authors read and approved the final manuscript.

## Conflict of Interest Statement

The authors declare that the research was conducted in the absence of any commercial or financial relationships that could be construed as a potential conflict of interest.

## Supplementary Material

FIGURE S1 **Flowchart for literature search**.
