# Supplementary figures and images for: The Acute Relationships Between Affect, Physical Feeling States, and Physical Activity in Daily Life: A Review of Current Evidence
Source: Front Psychol. 2015 Dec 23;6:1975. doi: 10.3389/fpsyg.2015.01975 (PMC4688389; doi:10.3389/fpsyg.2015.01975)

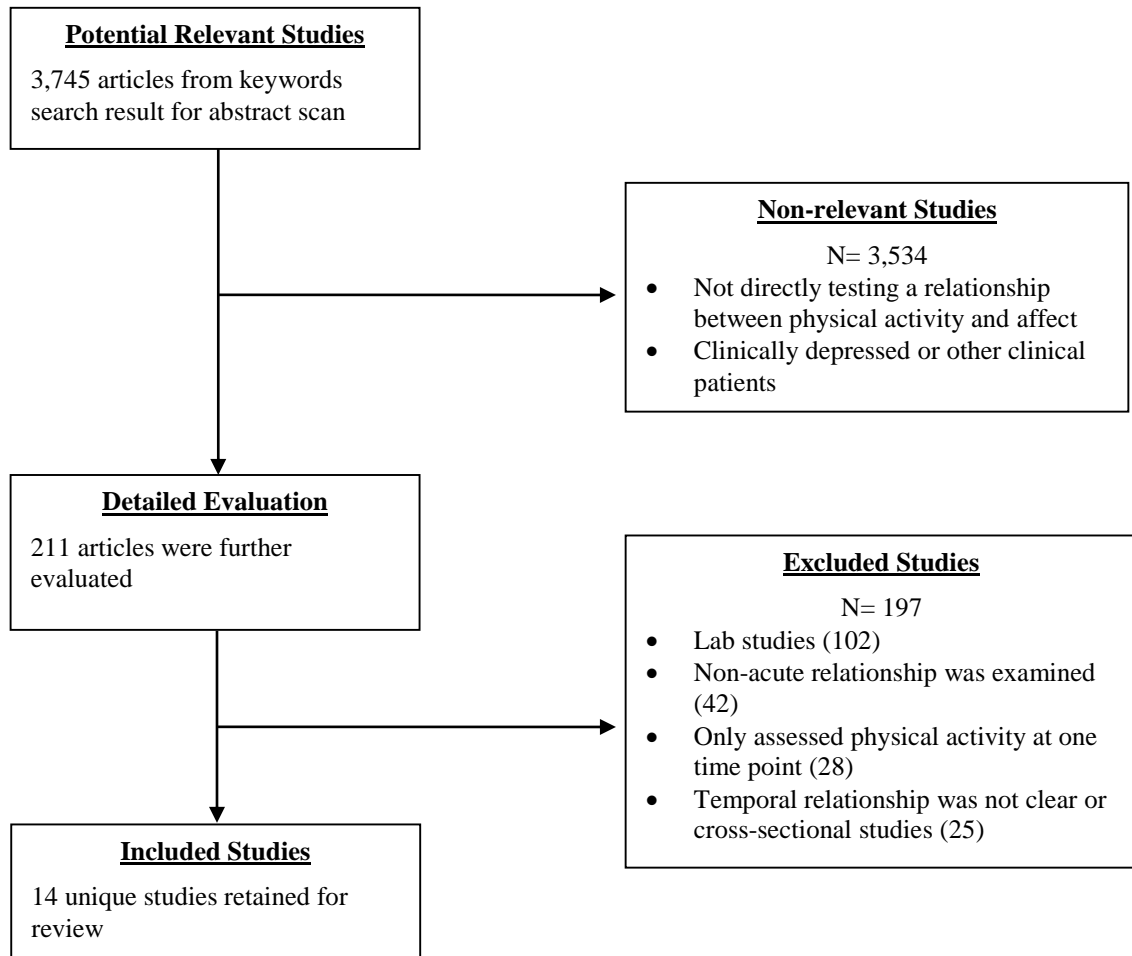

Supplement: FIGURE S1 — Flowchart for literature search. [file Image_1.PDF]
