# Supplementary material for: The Acute Relationships Between Affect, Physical Feeling States, and Physical Activity in Daily Life: A Review of Current Evidence
Source: Front Psychol. 2015 Dec 23;6:1975. doi: 10.3389/fpsyg.2015.01975 (PMC4688389; doi:10.3389/fpsyg.2015.01975)
Supplement: Supplementary file 2 [file Table_1.DOCX]

Table S1.

**OVID MEDLINE Search Strategy**

Completed on November 2, 2015

| 1. physical activity.mp. |
| --- |
| 2. exercise.mp. or Exercise/ |
| 3. 1 or 2 |
| 4. mood.mp. or Affect/ |
| 5. emotion.mp. or Emotions/ |
| 6. 4 or 5 |
| 7. 3 and 6 |
| 8. limit 7 to english language |
| 9. limit 8 to humans |
